# MwdpNet: towards improving the recognition accuracy of tiny targets in high-resolution remote sensing image

**DOI:** 10.1038/s41598-023-41021-8

**Published:** 2023-08-24

**Authors:** Dongling Ma, Baoze Liu, Qingji Huang, Qian Zhang

**Affiliations:** School of Surveying and Geo-Informatics, Shandong Jianzhu University, Jinan, 250101 China

**Keywords:** Engineering, Mathematics and computing

## Abstract

This study aims to develop a deep learning model to improve the accuracy of identifying tiny targets on high resolution remote sensing (HRS) images. We propose a novel multi-level weighted depth perception network, which we refer to as MwdpNet, to better capture feature information of tiny targets in HRS images. In our method, we introduce a new group residual structure, S-Darknet53, as the backbone network of our proposed MwdpNet, and propose a multi-level feature weighted fusion strategy that fully utilizes shallow feature information to improve detection performance, particularly for tiny targets. To fully describe the high-level semantic information of the image, achieving better classification performance, we design a depth perception module (DPModule). Following this step, the channel attention guidance module (CAGM) is proposed to obtain attention feature maps for each scale, enhancing the recall rate of tiny targets and generating candidate regions more efficiently. Finally, we create four datasets of tiny targets and conduct comparative experiments on them. The results demonstrate that the mean Average Precision (mAP) of our proposed MwdpNet on the four datasets achieve 87.0%, 89.2%, 78.3%, and 76.0%, respectively, outperforming nine mainstream object detection algorithms. Our proposed approach provides an effective means and strategy for detecting tiny targets on HRS images.

## Introduction

Target detection in high-resolution remote sensing (HRS) images is currently an important area of research for intelligent interpretation of remote sensing images. Accurately identifying tiny targets in HRS images is the primary task of remote sensing image target detection^1–5^. However, tiny targets in HRS images occupy only a few pixels, have indistinct features, and are easily affected by background interference. These factors make it difficult for existing network detection models to extract sufficient semantic information for these targets, resulting in poor detection and recognition performance and significant limitations. Therefore, detecting tiny targets in HRS images remains a significant challenge.

The emergence of deep learning^6–8^ has provided an automated framework for feature extraction and representation, including classification and targets detection^9–12^. Currently, widely used deep learning detection algorithms can be mainly divided into two categories. The first category is two-stage targets detection methods. Since the proposal of Region-convolutional neural network (R-CNN) by Ross Girshick et al.^13^, improved algorithms based on R-CNN have emerged successively, such as Fast R-CNN^14^, Faster R-CNN^15^, Mask R-CNN^16^, etc. These algorithms divide the targets detection process into two stages. Firstly, they determine the targets region and extract the feature information of candidate region targets. Then, they classify and recognize the regions using CNN to further predict and identify the position and category of candidate targets. The second category is one-stage targets detection algorithms, such as Wei Liu et al.'s SSD^17^ and Joseph Redmon et al.'s YOLO^18^. Currently, the latest algorithms in the SSD series mainly include RSSD^19^ and FFESSD^20^, and the YOLO series include YOLOV4^21^, YOLOV5^22^, and YOLOV6-M^23^ versions. These methods directly predict the position and category of the target through the network, so they have a faster detection speed.

Targets detection has always been a research topic, but the problem of detecting tiny targets has been largely overlooked. Existing deep learning-based targets detection techniques have mainly focused on four approaches: (1) Changing backbone network. For instance, densely connected convolutional network (DenseNet)^24^ and Scale-Transferrable Object Detection (STDN)^25^. (2) Increase receptive field. For example, the RFB module based on the Inception algorithm structure^26,27^ and the TridentNet algorithm based on the ResNet-101 network^28,29^. (3) Feature fusion. For example, the NAS-FNP feature pyramid structure^30^. (4) Cascade networks, such as the R-FCN^31^ algorithm and the NetAdapt^32^ algorithm. However, existing mainstream deep learning networks still have some problems in detecting tiny targets on HRS images. For example, the backbone network is difficult to effectively extract tiny target feature information, the tiny targets on an image are small in scale and can be easily scrambled or occluded, and the semantic feature information of the shallow feature map of tiny targets is weak.

Based on the above discussion and challenges of detecting tiny targets in HRS images, including the difficulty of effectively extracting target feature information, small target size, and weak feature response, we propose an effective framework called Multi-level Weighted Depth Perceptions Network (MwdpNet). This framework aims to address these challenges and improve the detection of tiny targets in HRS images. Our proposed MwdpNet uses a multi-level feature weighted fusion strategy to adaptively process semantic and analytical features of tiny targets, gradually restoring their edge information. The proposed MwdpNet also includes a deeper perception module and a channel attention guidance module to capture contextual information. The effectiveness and universality of our proposed MwdpNet are validated through experiments and comparisons with mainstream networks using a self-built dataset.

The main contributions of this paper are summarized as follows:

- We propose a framework for detecting tiny targets in HSR images, called MwdpNet, which combines low-level semantic information, high-level semantic information, multi-level feature information, and context-aware information. Furthermore, we construct a new grouped residual structure and propose a backbone-enhanced network, called S-Darknet53.
- A multi-level feature weighting and fusion strategy is proposed to combine shallow features from different layers, resulting in enhanced semantic features that enable the network to focus more on important semantic information of tiny targets. Additionally, the framework allows for the adaptive selection of high-quality training instances to stabilize model training and achieve accurate regression of tiny targets at various scales.
- To better represent features, a deeper perception module (DPModule) is proposed, which performs similar scale averaging on the enhanced shallow semantic features and converts the resulting convolutional layer features into vectors. To prevent the problem of dimensionality disaster caused by high-dimensional vectors, Principal Component Analysis dimensionality reduction algorithm is used. The reduced shallow enhanced features are then fused with deep features to form new vector features, which can express more rich semantic information of tiny targets.
- A channel attention guidance module (CAGM) is proposed to enhance multi-level features and multi-scale contextual representations. The CAGM employs multi-layer perceptrons to focus more on the positional sensitivity of HRS images.

## Methods

**Figure 1:**
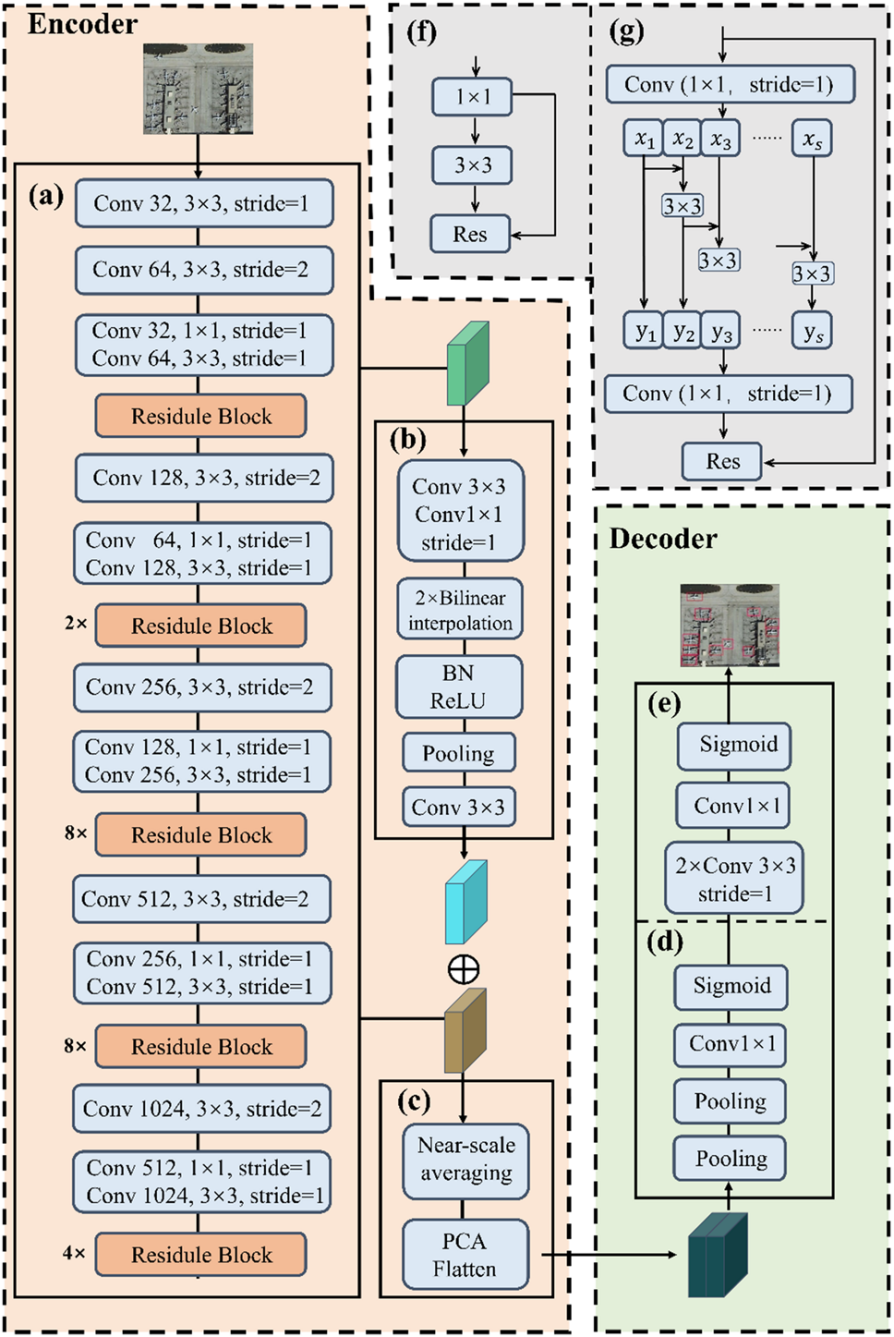
The framework and modules of the proposed MwdpNet. (a) Backbone network, (b) Shallow Enhancement Component for multi-level feature fusion, (c) DPModule, (d) CAGM, (e) Pixel Classifier, (f) Original Residule Block, (g) Grouped Residual Structure for network widening.

The overall structure of our proposed MwdpNet framework is illustrated in Fig. 1. MwdpNet consists of a single-stream encoder and decoder. The former extracts feature from the input image to obtain low-level features, while the latter fuses the processed feature maps. During the training process, the parameters are iteratively updated by minimizing the loss between the forward output and the reference output. (1) Encoder: In this paper, the input HRS image is feature-extracted through the encoder to obtain low-level features. It mainly consists of three core components: (a) backbone enhancement network; (b) shallow enhancement module; (c) DPModule. This section mainly introduces the backbone enhancement network, while other parts will be introduced in later sections. (2) Decoder: It consists of the Channel Attention Guided Module (CAGM) (Fig. 1d) and the pixel classifier (Fig. 1e). The CAGM module employs channel attention to boost the representation of multi-level features. Initially, the low-level features derived from the improved backbone network undergo max pooling and average pooling operations along the channel dimension. The resulting two channels are fused via channel fusion, yielding new features. These new features, along with the high-level features, are then refined by the CAGM module. Ultimately, the classifier produces a binary image.

### Backbone enhancement network

We improve the original residual network structure of Darknet-53 to form a new backbone enhancement network, S-Darknet53. We borrow the structure of Res2Net^33^ to replace the original residual structure with grouped residuals. The original residual network structure is shown in Fig. 1f, and the improved grouped residual structure is shown in Fig. 1g. Compared to Res2Net, S-Darknet53 has faster detection with equal prediction accuracy. Meanwhile, S-Darknet53 effectively improves the extraction of the tiny targets' features and enhances the table capability of tiny targets compared to Darknet-53. Each grouped residual structure is down-sampled by a 3 × 3 convolutional layer with a stride of 2 between them. In the grouped residual structure, the 3 × 3 convolution in the residual block is replaced by smaller convolutions grouped into s channels (${x}_{1},{x}_{2},{x}_{3}$*,….*${x}_{s}$), where each channel group has the same width and height, and the number of channels in each group is 1/s of the input feature map. The computation process for each group is as follows:

1 $$ y_{i} = \left\{ {\begin{array}{ll} {x_{i} ,} & \quad  {i = 1} \\ {Conv_{3 \times 3} \left( {x_{i} + y_{i - 1} } \right),} &  \quad  {1 < i < s} \\ \end{array} } \right. $$

where ${Conv}_{3\times 3}$ is a 3 × 3 convolutional kernel, and $s$ is the scale control parameter. By interweaving the feature information of different channels in the same layer of convolutional layers and connecting unused channels, this approach significantly improves the utilization of channel feature information in the backbone network and enhances its feature extraction ability to obtain more fine-grained features of tiny targets.

### Multi-level feature weighted fusion

**Figure 2:**
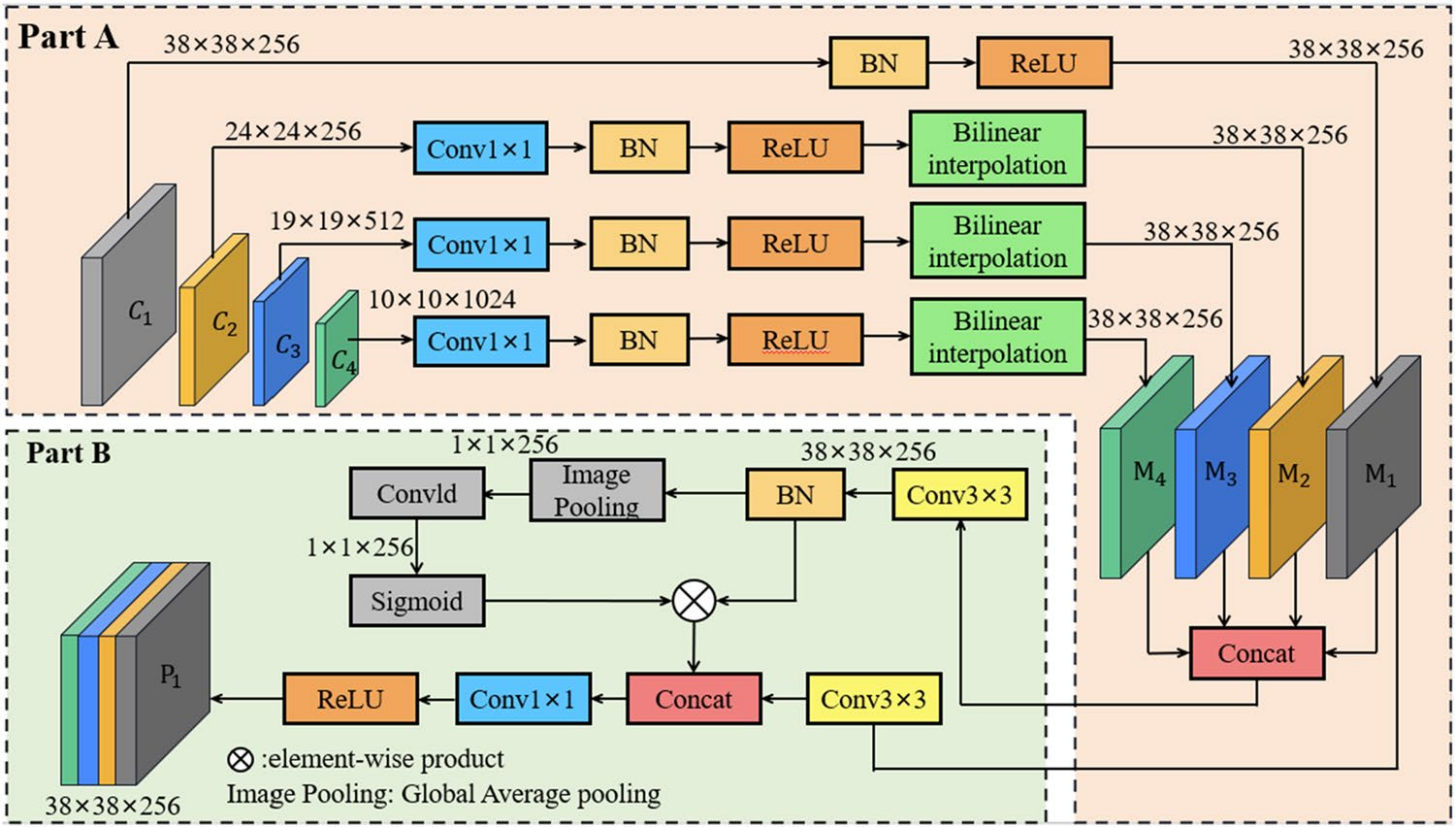
Multi-level feature weighted fusion strategy. It is divided into Part A and Part B, which can adaptively add weights to channels.

We propose a multi-level feature weighted fusion strategy to further enhance the network's ability to recognize tiny targets, as shown in Fig. 2. In the deep learning-based image salient targets detection feature network, low-level features have precise details and edge information about the target, and fusing low-level features at different levels of the network can improve the performance of target detection. Firstly, the image is fed into the backbone enhancement network to output different levels of feature maps $\mathrm{C}=\left\{{\mathrm{C}}_{1},{\mathrm{C}}_{2},{\mathrm{C}}_{3},{\mathrm{C}}_{4}\right\}$. ${\mathrm{C}}_{1}$ is processed through BN and ReLU to obtain the ${\mathrm{M}}_{1}$ feature map with constant size channels. Then the number of channels of ${\mathrm{C}}_{2}$ is reduced by 1 × 1 convolution, and up-sampling is performed through BN, ReLU and bilinear interpolation to obtain ${\mathrm{M}}_{2}$, which has different dimensions from ${\mathrm{C}}_{2}$, and the above steps are repeated to obtain ${\mathrm{M}}_{3}$, ${\mathrm{M}}_{4}$ feature maps. The main idea of the proposed strategy is to weight different dimensional channels of the base fusion feature map, thus selecting the important feature information of the fusion feature map. This enables the fusion of semantic features and early features to be more effective, and allows the semantic information of the shallow convolutional layers to be fully enhanced.

### Deeper perception module

**Figure 3:**
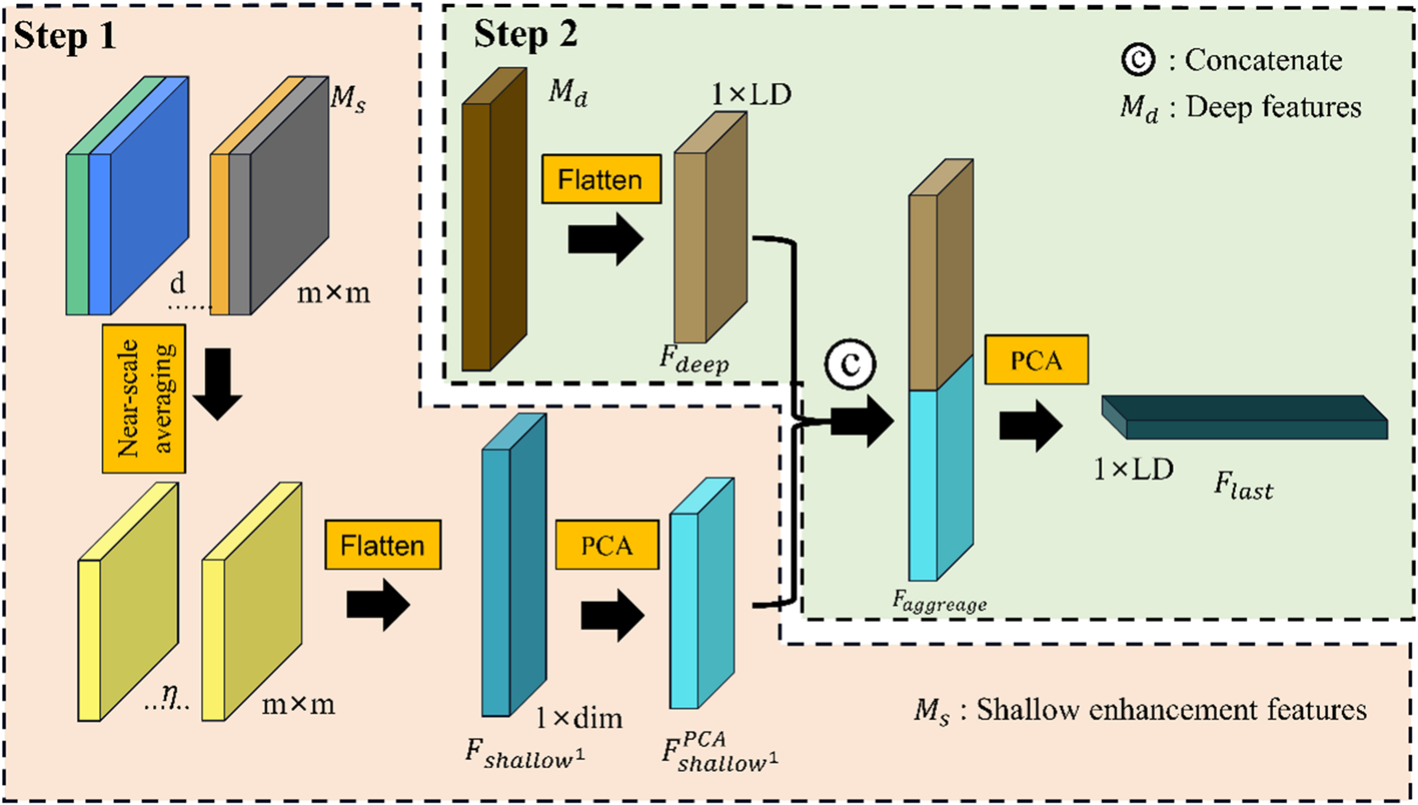
Processing flow of the deep perception module. Step 1 is the nearby scale averaging, and Step 2 is the enhancement of shallow features.

The deeper perception module proposed in this paper mainly deals with the shallow enhancement features obtained in the previous section and the deep features. Figure 3 shows the overall processing of the Deeper Perception Module (DPModule). The DPModule mainly consists of two steps. (1) Nearby scale averaging: the shallow enhancement features obtained in the previous section and the adjacent convolution layers are integrated, flattened into a feature vector, and reduced by Principal Component Analysis. (2) Fusion of shallow enhancement feature vector and deep feature vector: the dimensionality-reduced shallow enhancement feature vector and deep feature vector are cascaded from top to bottom to form a new dense feature vector.

### Channel attention guided module

**Figure 4:**
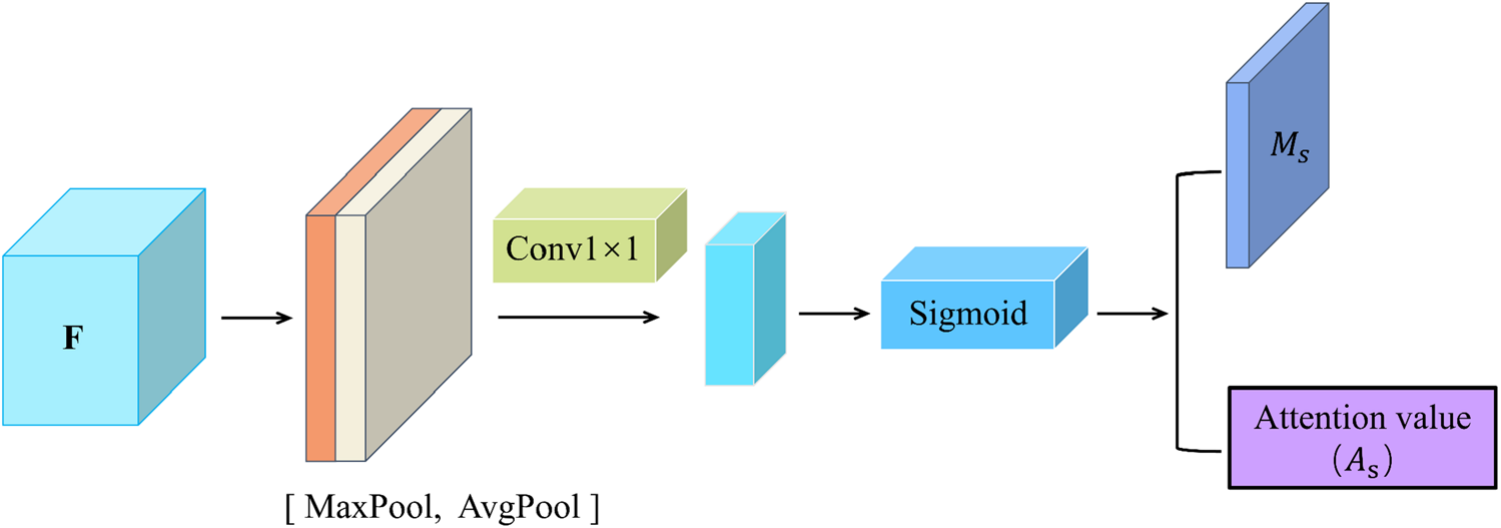
Channel attention guidance module CAGM. Obtaining ${\mathrm{M}}_{\mathrm{s}}$ and ${\mathrm{A}}_{\mathrm{S}}$ by two types of pooling and one convolution after classification.

The Channel Attention Guidance Module (CAGM) proposed in this paper is an important module in our proposed MwdpNet network. CAGM focuses on location information and is designed to enhance the representation ability of multi-level features in HRS images. The design of the CAGM module is shown in Fig. 4.

The features obtained from the backbone network at each scale, denoted as $F$. Firstly, the acquired features $F$ are pooled along the channel direction by maximum pooling and mean pooling, and then these two channels are merged using channel fusion to obtain new features. Immediately after that, a $1\times 1$ convolution is applied on the merged fused features with the multi-classification function Sigmoid to finally generate the spatial attention map ${M}_{s}$, which can be expressed as:

2 $$ \begin{aligned} M_{s} \left( F \right) & = \sigma \left( {f^{1 \times 1} \left( {\left[ {AvgPool\left( F \right);MaxPool\left( F \right)} \right]} \right)} \right) \\ &=\sigma \left({f}^{1\times 1}\left(\left[{F}_{avg}^{S};{F}_{max}^{S}\right]\right)\right) \end{aligned}$$

where, ${F}_{avg}^{S}\in {\mathbb{R}}^{1\times \mathrm{H}\times \mathrm{W}}$ and ${F}_{max}^{S}\in {\mathbb{R}}^{1\times \mathrm{H}\times \mathrm{W}}$ respectively represent average pooling and max pooling along the channel dimension. ${\mathrm{f}}^{1\times 1}$ represents a 1 × 1 filter used in the convolution operation.

### Loss function optimization

Due to the difficulty in classifying hard samples between tiny targets and background, the model may suffer from the problem of imbalanced positive and negative samples. Therefore, it is necessary to consider the contribution ratio of different samples to the loss and give more weight to tiny targets samples in the loss function. In this paper, the loss function is defined as:

3 $$ L\left( {p_{t} } \right) = - \beta_{t} \left( {1 - p_{t} } \right)^{\varepsilon } \log \left( {p_{t} } \right) $$

where, the variable represents the probability that a sample belongs to the true label. The formula for calculating is:

4 $${p}_{t}=\left\{ {\begin{array}{ll} {p} & \quad  { if \;\; y=1} \\ {1-p} &  \quad  { otherwise } \\ \end{array} } \right.$$

during the model training process, there is often a significant difference in the number of positive and negative samples, which can lead to imbalanced contribution to the total loss. To address this issue, a factor $\beta \in \left[0,1\right]$ is designed to control the weight of positive and negative samples on the total loss, in order to balance the contribution of different samples to the total loss. In addition, an adaptive modulation factor ${\left(1-{p}_{t}\right)}^{\varepsilon }$ is added to optimize the easy and difficult-to-classify samples. Here, $\upvarepsilon \ge 0$ is a focusing parameter, and the modulation factor ${\left(1-{\mathrm{p}}_{\mathrm{t}}\right)}^{\upvarepsilon }$ can reduce the weight of the loss for easy-to-classify samples and increase the weight of the loss for difficult-to-classify samples. In this paper, the tiny target recognition algorithm is trained with $\beta =0.25$ and $\varepsilon =2$ to achieve the best detection of the model.

## Experimental results

### Dataset description

Dataset 1: Based on the DOTA dataset^34^, we selected small vehicles, boats, and airplanes as tiny targets. We manually selected a total of 1022 images containing small vehicles and airplane categories, with small vehicles ranging in size from 24.7 × 24.7 to 40.9 × 40.9, and airplanes ranging from 37.1 × 37.1 to 51.2 × 51.2. Dataset 2: Based on the VEDAI dataset^35^, we merged the smallest sized vehicles including cars, campers, trucks, and lorries into one category, and selected targets ranging in size from 11.5 × 11.5 to 18.7 × 18.7. Dataset 3: Based on the VEDAI dataset, we evaluated the detection performance of 9 target categories (boats, cars, campers, airplanes, shuttles, tractors, trucks, cargo trucks, and other categories). The displayed target sizes range from 13.5 × 13.5 to 24.9 × 24.9. Dataset 4: Based on the NWPU VHR-10 dataset^36^, we manually selected 526 images containing airplanes, boats, tanks, and vehicles. The displayed target sizes range from 42.28 × 42.28 to 48.32 × 48.32, creating a tiny targets dataset. The details of each data set are shown in Table 1.

**Table 1:** The four experimental data sets constructed in this paper.

Datasets	Data sources	Input image size	Tiny targets	Tiny target size	Number of targets
Datasets1	DOTA	1000 × 1000 2000 × 2000	Small vehicle, ship, plane	24.7 × 24.7–51.2 × 51.2	63,070
Datasets2	VEDAI	1024 × 1024 512 × 512	Car, pick-up, van	11.5 × 11.5–18.7 × 18.7	2108
Datasets3	VEDAI	1024 × 1024	Boat, camping, car, others, pickup, tractors, truck, vans, plane	13.5 × 13.5–24.9 × 24.9	3640
Datasets4	NWPU VHR-10	–	Plane, ship, storage tank, vehicle	42.2 × 42.2–48.3 × 48.3	2078

### Details of experiments

The experiment was conducted using Python 3.6 and Pytorch 1.2. The CPU model was i7-10875H with 32 GB memory, and two NVIDIA GeForce RTX 3060 GPUs were used. The stochastic gradient descent algorithm was used to update and optimize the network model's weights during training. Two scales were used in training datasets 1–4, with a batch size of 16, initial learning rate of 0.001, learning rate decay weight of 0.0005, momentum factor of 0.99, and maximum iteration set to 40,000 (approximately 40 epochs). Additionally, to ensure training stability, a warm-up process with a small learning rate (1 × ${\mathrm{e}}^{-6}$) was used for the first 300 iterations, followed by a change to 0.001. The learning rate was reduced to 1/10 at iterations 10,000, 20,000, and 30,000. Four experiments were conducted to evaluate and compare the performance of the proposed network with other mainstream algorithms for targets detection.

### Evaluation metrics

To quantitatively evaluate the performance of our proposed MwdpNet, we use Average Precision (AP), mean Average Precision (mAP), Precision-Recall Curve (PRC), F1 score, and Intersection over Union (IoU) as the evaluation indicators for the network. AP is the ratio of the number of correct identifications to the total number of identifications. mAP is used to indicate the accuracy of all target detections, i.e. the average of all AP. Recall is the ratio of the number of correct identifications to the total number of marks. F1 score combines Ap and Recall to measure the performance of the network in a comprehensive way. IoU is used to indicate the overlap rate of candidate and marker boxes. All the indicators are calculated as follows:

5 $$\begin{array}{c}Precision=\frac{TP}{TP+FP}\end{array}$$

6 $$\begin{array}{c}Recall=\frac{TP}{TP+FN}\end{array}$$

7 $$\begin{array}{c}F1=\frac{2}{\left(\frac{1}{Precision}\right)+\left(\frac{1}{Recall}\right)}\end{array}$$

8 $$\begin{array}{c}AP={\int }_{0}^{1}P\left(r\right)dr\end{array}$$

9 $$\begin{array}{c}IoU=\frac{TP}{TP+FP+FN}\end{array}$$

where TP is true positive, TN is true negative, FP is false positive, and FN is true negative, P(r) is PRC.

### Experiment 1

The performance of MwdpNet was evaluated by inputting two sizes of images, denoted as "*m*" for 1000 × 1000 images and "*l*" for 2000 × 2000 images (e.g., SSD_m_: inputting 1000 × 1000 images into SSD). The compared algorithms were single-stage detection algorithms (SSD^17^, RSSD^19^, FFESSD^20^, MDSSD^37^). The backbone network used by SSD is VGG16, and ResNet-101 is used by RSSD, FFESSD and MDSSD. The results are shown in Table 2.

**Table 2:** Detection results of various detection algorithms in Experiment 1 on Dataset 1.

Method	Precision	Recall	F1-score	IoU	mAP
SSD_*m*_^17^	0.536	0.745	0.790	0.803	0.794
RSSD_*m*_^19^	0.626	0.782	0.809	0.879	0.796
FFESSD_*m*_^20^	0.868	0.876	0.851	0.853	0.823
MDSSD_*m*_^37^	0.869	0.874	0.905	0.902	0.829
Ours_*m*_	**0.875**	**0.881**	**0.910**	**0.910**	**0.831**
SSD_*l*_^17^	0.452	0.754	0.823	0.823	0.834
RSSD_*l*_^19^	0.787	0.791	0.879	0.860	0.843
FFESSD_*l*_^20^	0.783	0.846	0.840	0.861	0.859
MDSSD_*l*_^37^	**0.872**	0.888	0.900	0.891	0.863
Ours_*l*_	0.851	**0.890**	**0.912**	**0.899**	**0.870**

Significant values are in bold.

### Experiment 2

Based on extensive experience, the authors found that YOLO series algorithms perform well on the VEDAI dataset, and thus compared our model with them. We conducted comparative experiments with two different input sizes (512 × 512 and 1024 × 1024) and single-stage detection algorithms (YOLOV4^21^, YOLOV5^22^, YOLOV6-M^23^). The backbone network used by YOLOV4, YOLOV5 and YOLOV6-M is Darknet-53. The results are shown in Table 3.

**Table 3:** Detection results of various detection algorithms in Experiment 2 on Dataset 2.

Method	Precision	Recall	F1-score	IoU	mAP
YOLOV4_*m*_^21^	0.664	0.819	0.723	0.571	0.773
YOLOV5_*m*_^22^	0.729	0.820	0.792	0.635	0.785
YOLOV6-M_*m*_^23^	**0.849**	**0.890**	**0.869**	0.752	**0.880**
Ours_*m*_	0.836	0.886	0.860	**0.755**	0.872
YOLOV4_*l*_^21^	0.601	0.711	0.660	0.501	0.751
YOLOV5_*l*_^22^	0.721	0.827	0.762	0.673	0.760
YOLOV6-M_*l*_^23^	**0.864**	0.879	**0.874**	**0.790**	**0.896**
Ours_*l*_	0.858	**0.889**	0.873	0.775	0.892

Significant values are in bold.

### Experiment 3

To comprehensively evaluate the effectiveness of our network, we compared it with two-stage detection algorithms in contrast to single-stage algorithms in experiments 1 and 2. Two-stage algorithms are better at identifying tiny targets, so we conducted this experiment on dataset 3. The input image size for experiment 3 was set to 1024 × 1024. The compared algorithms include Faster-RCNN^15^, OHEM^38^, ION^39^, and R-FCN^31^. The backbone network used by Faster-RCNN, OHEM and ION is VGG16, and ResNet-101 is used by R-FCN. The results are shown in Table 4.

**Table 4:** Detection results of various detection algorithms in Experiment 3 on Dataset 3.

Method	AP									mAP
	Car	Boat	Camping	Plane	Vans	Truck	Tractors	Pickup	Others	
Faster^15^	0.676	0.423	0.517	0.799	0.615	0.622	0.742	0.713	0.752	0.651
OHEM^38^	0.719	0.583	0.767	0.809	0.707	0.688	0.773	0.779	0.792	0.735
ION^39^	0.764	0.579	0.779	0.843	0.724	0.739	0.807	0.802	0.823	0.762
R-FCN^31^	0.846	0.583	**0.817**	0.869	**0.775**	0.722	0.742	0.773	0.812	0.771
Ours	**0.885**	**0.588**	0.789	**0.875**	0.763	**0.761**	**0.806**	**0.821**	**0.819**	**0.783**

Significant values are in bold.

### Experiment 4

**Figure 5:**
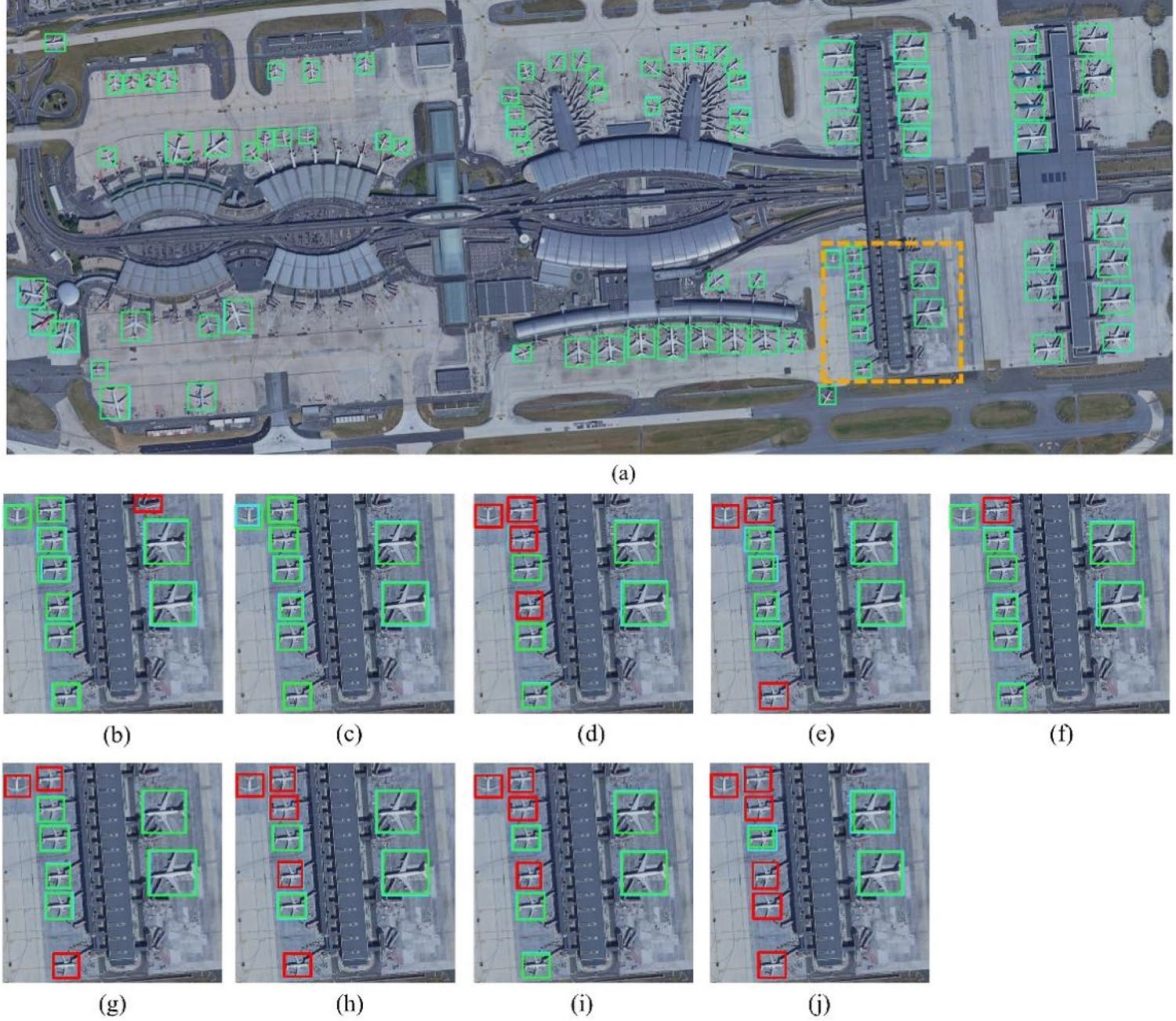
Experiment 4 on Dataset 4. Comparison of different algorithms: (**a**) MwdpNet, (**b**) YOLOV5, (**c**) YOLOV6-M, (**d**) RSSD, (**e**) FFESSD, (**f**) MDSSD, (**g**) Faster, (**h**) OHEM, (**i**) ION, (**j**) R-FCN. The light blue boxes represent ground truth, green boxes represent predicted boxes, and red boxes represent malfunction detection boxes.

Experiment 4 was conducted on Dataset 4. The compared algorithms include single-stage detection algorithms such as RSSD, FFESSD, MDSSD, YOLOV5, and YOLOV6-M, and two-stage detection algorithms such as Faster-RCNN, OHEM, ION, and R-FCN. The results are shown in Table 5, where AP_s_, AP_m_, and AP_l_ in the table represent small targets (area < 32^2^), medium targets (area < 96^2^), and large targets (area > 96^2^), respectively. Visual contrast is shown in Fig. 5. The orange box is a partial area that we have chosen for the comparative experiment. The backbone used by RSSD, FFESSD, MDSSD and R-FCN is ResNet-101. YOLOV5 and YOLOV6-M use Darknet-53 for the backbone, and Faster-RCNN, OHEM and ION use VGG16 for the backbone. The light blue boxes represent ground truth, green boxes represent predicted boxes, and red boxes represent malfunction detection boxes.

**Table 5:** Detection results of various detection algorithms in Dataset 4 in Experiment 4.

Method	Precision	Recall	F1-score	IoU	mAP	AP_s_	AP_m_	AP_l_
Two-stage								
Faster^15^	0.489	0.629	0.550	0.379	0.619	–	–	–
OHEM^38^	0.503	0.715	0.604	0.419	0.626	0.281	0.439	0.652
ION^39^	0.517	0.637	0.632	0.391	0.636	0.267	0.444	0.683
R-FCN^31^	0.521	0.727	0.677	0.436	0.692	0.302	0.525	0.707
One-stage								
RSSD^19^	0.542	0.681	0.580	0.432	0.698	0.291	0.591	0.721
FFESSD^20^	0.700	0.772	0.709	0.792	0.742	0.351	0.665	0.752
MDSSD^37^	0.704	0.744	0.718	0.801	0.744	0.359	0.581	0.779
YOLOV5^22^	0.723	0.781	0.723	0.809	0.741	0.310	0.585	0.780
YOLOV6-M^23^	**0.781**	0.722	**0.819**	**0.821**	**0.764**	0.382	**0.598**	**0.796**
Ours	0.775	**0.781**	0.810	0.812	0.760	**0.393**	0.585	0.789

Significant values are in bold.

### Model complexity comparison

Table 6 shows the comparison of the model complexity of our proposed MwdpNet and comparison networks, including parameters, computations, and training time. VGG16 has the most parameters (138.7 M) and computations (15.47GFLOPS/s), resulting in longer training time (251, 223, 310). Our proposed MwdpNet has 73.2 M parameters, larger than Darknet-53 (40.6 M) and ResNet-101 (44.0 M), but with no significant difference in computation. MwdpNet has training times of 180 s (dataset 1), 196 s (dataset 2), and 171 s (dataset 3), which is similar to traditional models and much lower than VGG16. Therefore, our proposed MwdpNet achieves the best detection performance without using too much time and cost, indicating that the network can effectively balance computation and recognition efficiency.

**Table 6:** Comparison of the complexity of the proposed model with other models.

Model	Parameters (M)	Computation (GFLOPS/s)	Dataset1 training (s)/epoch	Dataset2 training (s)/epoch	Dataset3 training (s)/EPOCH
VGG16	138.7	15.47	251	223	310
ResNet-101	44.0	10.39	104	156	120
Res2Net-50	34.5	12.46	143	179	167
Tiny-darknet	16.9	11.34	178	179	210
Darknet-53	40.6	12.57	129	187	175
MwdpNet	73.2	13.23	180	196	171

### Ablation study for the proposed MwdpNet

**Figure 6:**
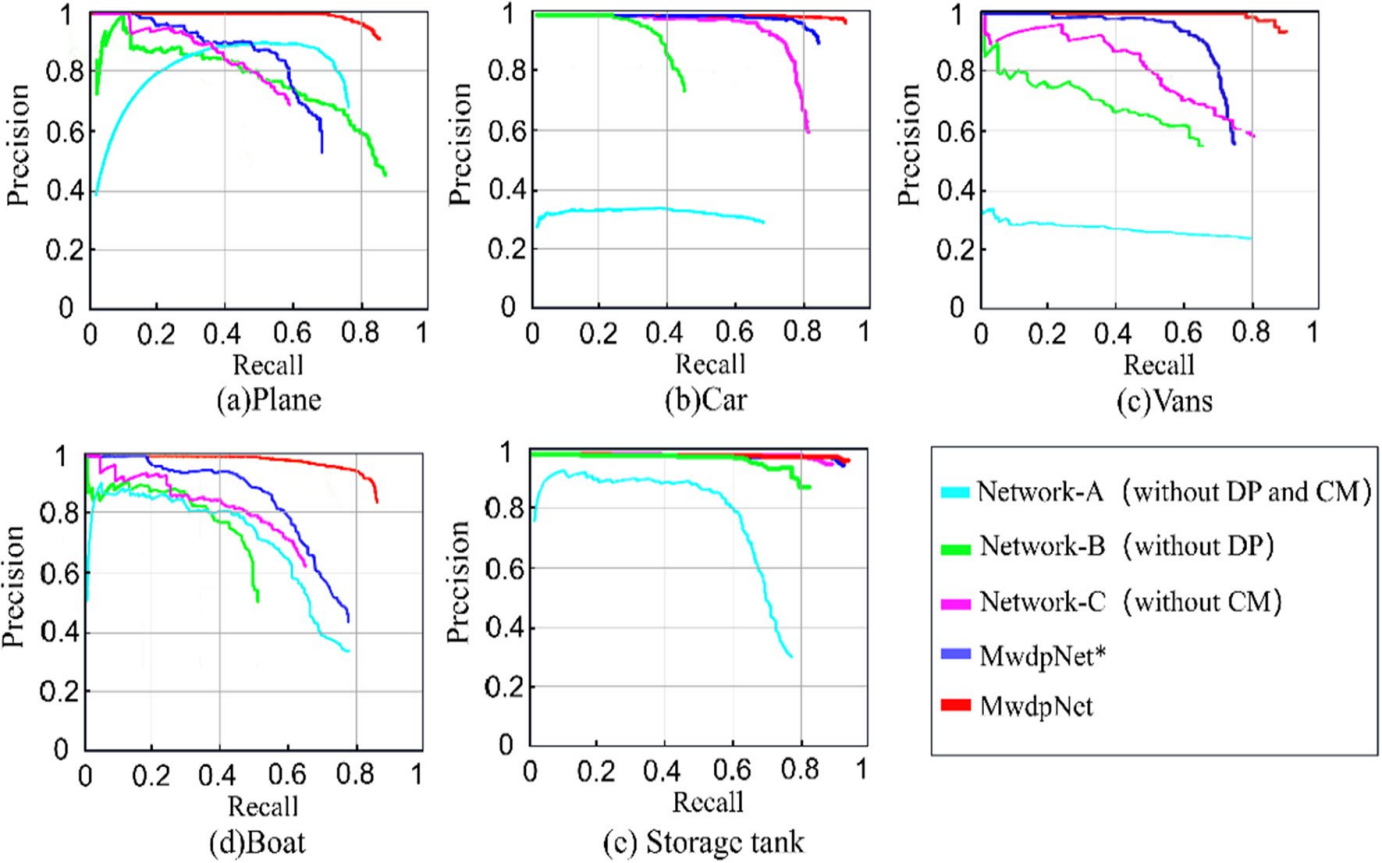
Precision-Recall curves (PRCs) of the proposed MwdpNet algorithm and comparison methods.

**Figure 7:**
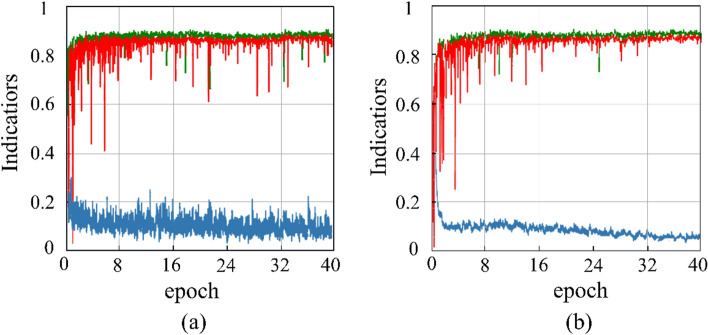
Two network validation curves. (**a**) Network-A, (**b**) MwdpNet. The blue, green and red lines represent Loss, F1 and AP respectively.

In order to investigate the impact of DPModule, CAGM, and multi-level feature fusion strategy on the performance of MwdpNet, we conducted a series of ablation experiments in this study. We tested the modules and strategies on Dataset 2 and selected the five tiny targets for comparison using the quantized PRCs curves obtained during training, as shown in Fig. 7. To obtain the baseline performance, we designed Network-A with truncated Darknet and additional convolutional layers, where DP (DPModule) and CM (CAGM) were both removed. To analyze the impact of CM, we discarded the reconstruction network but retained the fusion part in Network-B. Network-C was implemented with truncated Darknet, additional convolutional layers, and DP, which was used to analyze the feasibility of enhancing shallow feature information for tiny targets recognition. MwdpNet* was implemented with truncated Darknet, additional convolutional layers, DP, and CM, but without using the multi-level feature fusion strategy. MwdpNet is the complete network proposed in this paper. From Fig. 6, it is apparent that DP and CM make the PRCs of MwdpNet smoother, and the multi-level feature fusion strategy can effectively extract features from tiny targets on HRS images in a more reasonable manner.

Table 7 shows the AP scores for four types of tiny targets and the mAP for each network. Without DP and CM, Network-A achieved a baseline mAP of 0.388. For Network-B and Network-C, DP and CM achieved 21.1% and 30.6% mAP gains, respectively. The effectiveness of the multi-level feature weighted fusion strategy was also confirmed by the gain in AP scores. MwdpNet achieved a 7.4% mAP gain compared to MwdpNet* and demonstrated the feasibility of the proposed strategy for identifying tiny targets.

**Table 7:** Structural comparison of MwdpNet in different configurations.

	Network-A (without DP and FM)	Network-B (without DP)	Network-C (without CM)	MwdpNet*	MwdpNet
Plane	0.373	0.645	0.664	0.757	**0.881**
Car	0.341	0.687	0.689	0.834	**0.896**
Vans	0.355	0.576	0.701	0.786	**0.852**
Boat	0.382	0.663	0.765	0.820	**0.869**
Storage tank	0.493	0.674	0.653	0.821	**0.891**
mAP	0.388	0.649	0.694	0.803	**0.877**

Significant values are in bold.

During training, we also visualized the loss, F1, and AP values for different networks, as shown in Fig. 7. As can be seen from Fig. 7, the loss curve for Network-A shows a less obvious descending trend and more fluctuations compared to that of MwdpNet. The F1 and AP values of MwdpNet outperform those of Network-A. This indicates that our proposed DP and CM can be more effective in obtaining information about tiny targets in the dataset.

**Figure 8:**
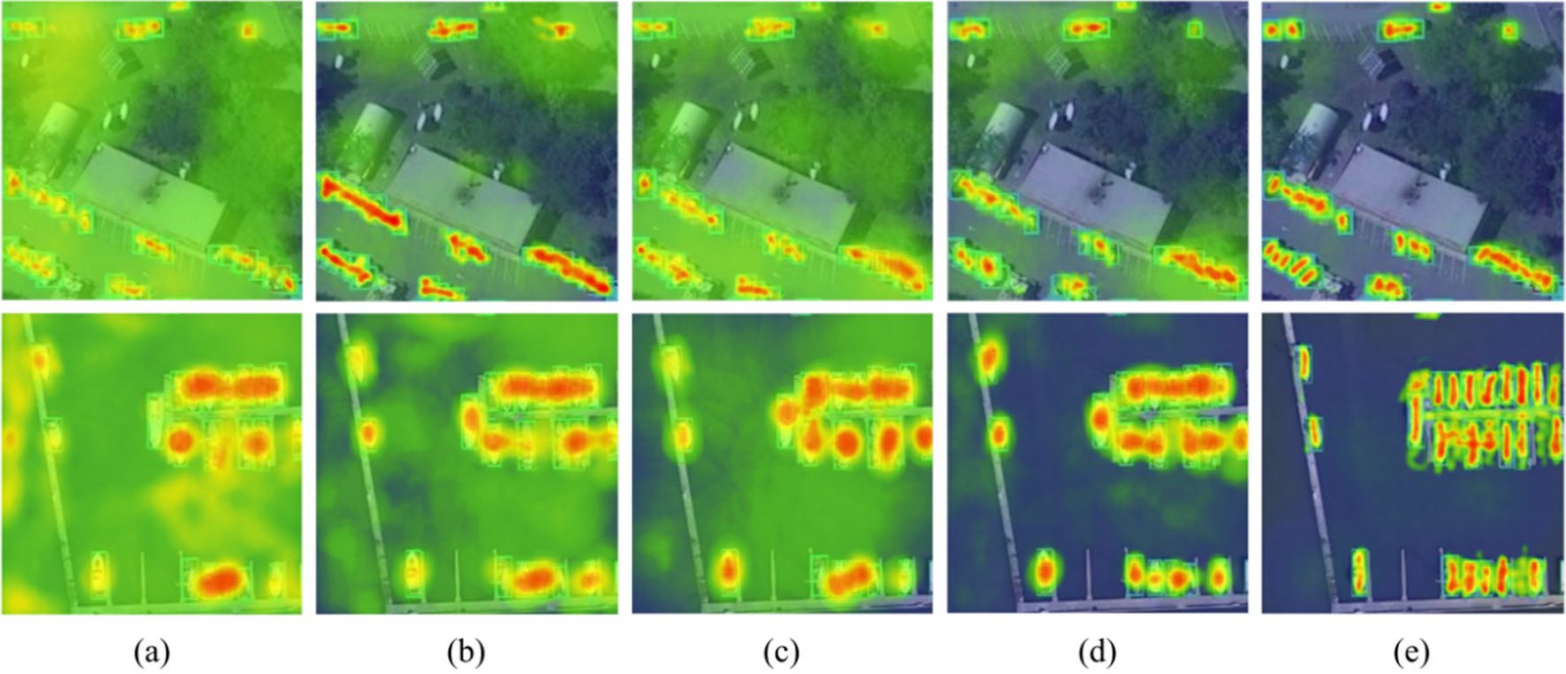
Shows the heatmaps of the shared feature maps after fusion for the five networks (**a**) Network-A, (**b**) Network-B, (**c**) Network-C, (**d**) MwdpNet*, and (**e**) MwdpNet.

Figure 8 shows the heatmaps of the five networks after feature fusion. For comparison, the heatmap area is cropped from a portion of the input image. The comparison of the heatmaps in (a)–(e) indicates that the response of the target areas in the shared feature maps is enhanced through DP, CM, and multi-level feature fusion, which is beneficial for improving the recognition ability of the network. Compared with (a), (b), and (c), the response of the background area is significantly weakened in (d) and (e), indicating that the target feature and localization accuracy have been improved. The background information can be fully utilized by CM, while the target feature can be more effectively extracted and fused by DP. Using the proposed modules and strategies can better focus the network on the target area.

## Discussion

We conduct a total of five experiments, including four comparative experiments and one ablation experiment. The four comparative experiments are designed to comprehensively evaluate the performance of our proposed MwdpNet and nine other mainstream algorithms for detecting tiny targets. The ablation experiment is performed to validate the effectiveness of the various modules of MwdpNet in detecting tiny targets.

By analyzing the four comparison experiments, we find that our proposed MwdpNet has several advantages in detecting tiny targets: (1) The proposed MwdpNet performs better in recognizing tiny targets in large-scale images. For example, in Experiment 1, Table 2 shows that when the input image is 2000 × 2000, the mAP of MwdpNet is 87.0%, which achieves the optimal detection accuracy and is 1.3% higher than MDSSD. In Experiment 2, Table 3 shows that when the input image is 1024 × 1024, the mAP of MwdpNet is 13.2% higher than that of YOLOV5, which is comparable to that of YOLOV6-M. (2) Our proposed MwdpNet is more capable of recognizing tiny targets with different shapes. In Experiment 3, as shown in Table 4, when recognizing targets of various shapes such as trucks, tractors, and cars, the APs of the proposed MwdpNet are 76.1%, 80.6%, and 88.5%, which are 3.9%, 6.4%, and 3.9% higher than that of the R-FCN, respectively. (3) The proposed MwdpNet can better recognize the edge information of tiny targets. As shown in the visual comparison image in Experiment 4 (Fig. 5), the bounding box predicted by MwdpNet can more accurately identify the location of tiny targets compared with the other nine mainstream algorithms.

In the ablation experiment, the precision-recall curves visualization (Fig. 6) for five tiny targets shows that our proposed MwdpNet is able to learn effectively. The heatmaps (Fig. 8) also indicate that the DPModule, CAGM, and multi-level feature fusion strategy of the proposed MwdpNet enhance the response of shared feature maps, which is beneficial for improving the accuracy of detecting tiny targets.

However, the precision advantage of the proposed MwdpNet is not particularly apparent when identifying tiny targets with fixed shapes and colors, such as planes and boats. For example, in Experiment 3, Table 4 shows that the mAP of our proposed MwdpNet for these targets are only 0.6% and 0.5% higher than those of R-FCN, respectively. This is because our proposed MwdpNet primarily focuses on exploring the contextual semantic feature information of tiny targets, which can better determine the position of tiny targets on the image. Therefore, in future work, we aim to improve the model's generalization ability while ensuring its performance in detecting tiny targets on large-scale images remains stable. We also plan to enhance the backbone network of the model and further accelerate its performance.

## Conclusion

In this paper, we presented a novel and effective MwdpNet framework for detecting tiny targets in HRS images. In order to improve the accuracy of tiny targets detection in HRS images, we have designed a multi-level feature weighted fusion strategy in the MwdpNet detection framework. This strategy fully utilizes feature maps of different sizes to enhance the detection performance of tiny targets and improves the residual structure to enhance the ability of feature channel information extraction in the backbone network. Additionally, the deep perception module (DPModule) and channel attention guidance module (CAGM) are introduced in MwdpNet to achieve good classification performance and improve the recall rate of tiny targets. The performance of the proposed MwdpNet has been evaluated on three public datasets, and ablation experiments have demonstrated the effectiveness of the proposed strategies and each module in MwdpNet, particularly for tiny targets.

This study reveals the possibility of fully extracting all semantic features contained in HRS images, and provides a more effective technical approach for exploring the spatial relationships and configurations between different feature units on images. This is the true significance of information extraction and target recognition in HRS images.

## Data Availability

All data generated or analyzed during this study are included in this published article.
